# Differentiation of brain metastases from small and non-small lung cancers using apparent diffusion coefficient (ADC) maps

**DOI:** 10.1186/s12880-021-00602-7

**Published:** 2021-04-15

**Authors:** Sebastian Johannes Müller, Eya Khadhraoui, Nicole E. Neef, Christian Heiner Riedel, Marielle Ernst

**Affiliations:** Department of Diagnostic and Interventional Neuroradiology, Georg-August-University Göttingen, Robert-Koch-Str. 40, 37075 Göttingen, Germany

**Keywords:** Brain metastases, Diffusion, SCLC, NSCLC, ADC

## Abstract

**Background:**

Brain metastases are particularly common in patients with small cell lung cancer (SCLC) and non-small cell lung cancer (NSCLC), with NSCLC showing a less  aggressive clinical course and lower chemo- and radio sensitivity compared to SCLC. Early adequate therapy is highly desirable and depends on a reliable classification of tumor type. The apparent diffusion coefficient is a noninvasive neuroimaging marker with the potential to differentiate between major histological subtypes. Here we determine the sensitivity and specificity of the apparent diffusion coefficient to distinguish between NSCLC and SCLC.

**Methods:**

We enrolled all NSCLC and SCLC patients diagnosed between 2008 and 2019 at the University Medical Center Göttingen. Cranial MR scans were visually inspected for brain metastases and the ratio of the apparent diffusion coefficient (ADC) was calculated by dividing the ADC measured within the solid part of a metastasis by a reference ADC extracted from an equivalent region in unaffected tissue on the contralateral hemisphere.

**Results:**

Out of 411 enrolled patients, we detected 129 patients (83 NSCLC, 46 SCLC) with sufficiently large brain metastases with histologically classified lung cancer and no hemorrhage. We analyzed 185 brain metastases, 84 of SCLC and 101 of NSCLC. SCLC brain metastases showed an ADC ratio of 0.68 ± 0.12 SD, and NSCLC brain metastases showed an ADC ratio of 1.47 ± 0.31 SD. Receiver operating curve statistics differentiated brain metastases of NSCLC from SCLC with an area under the curve of 0.99 and a 95% CI of 0.98 to 1, *p* < 0.001. Youden's J cut-point is 0.97 at a sensitivity of 0.989 and a specificity of 0.988.

**Conclusions:**

In patients with lung cancer and brain metastases with solid tumor parts, ADC ratio enables an ad hoc differentiation of SCLC and NSCLC, easily achieved during routine neuroradiological examination. Non-invasive MR imaging enables an early-individualized management of brain metastases from lung cancer.

*Trial registration*: The study was registered in the German Clinical Trials Register (DRKS00023016).

**Supplementary Information:**

The online version contains supplementary material available at 10.1186/s12880-021-00602-7.

## Background

The incidence of brain metastases in patients with lung cancer is approximately 20% [1]. Histologically, 38% of patients suffer from small cell lung cancer (SCLC), and in 62% non-small cell lung cancer (NSCLC) is found [2].

A reliable radiological marker for differentiating these subtypes could accelerate the start of therapy. A fast differentiation is essential for an efficient therapy, especially in case of SCLC. Today, biopsy and histological workup are essential before therapy is started. Beside surgical risks, wound healing can be insufficient under systemic therapy [3].

The relationship between diffusion restricted brain metastasis and underlying pathology is still a matter of debate [4]. There are a number of heterogeneous studies including very few patients and dealing with the value of diffusion imaging in patients with brain metastases, many of which even attempt to find a correlation to certain gene expressions [5]. Statistically, in solid tumor parts more cell membranes should be included in 1 cm^3^ of a small cell carcinoma than in NSCLC, because the cytoplasmic diameter is smaller in SCLC [6]. Thus, we hypothesize that the diffusion of water molecules should be less restricted in NSCLS compared to SCLC. We analyzed whether a differentiation of SCLC and NSCLC in brain metastases can be achieved with diffusion weighted imaging (DWI), i.e. the apparent diffusion coefficient (ADC) mapping. First evidence for an ADC based differentiation of histological subtypes of brain metastases from lung cancer stems from a most recent study [7]. However, time-consuming offline analyses restrict the implementation of this approach into everyday clinical routine. Here, we evaluated a practically feasible procedure that can be implemented in clinical routine and promote timely clinical decisions.

## Methods

### Study design

This was a retrospective analysis of a single-center non-interventional observational study. We followed the STARD 2015 protocol [8]. Institutional review board approval was obtained. The study was registered in the German Clinical Trial Register (No. DRKS00023016).

### Participants

We analyzed patients with pathologically confirmed SCLC or NSCLC and an MRI of the brain, regardless of the MRI manufacturer or the field strength. The study had to contain (1) a T1-weighted sequence with contrast agent (CA), (2) a T2-weighted sequence for detecting cystic parts and (3) a DWI Sequence with ADC mapping as calculated by the manufacturer.

The patients were checked, and excluded, if (1) no brain metastasis were found, (2) the size of the solid tumor parts were not large enough (< 0.25 cm^2^) for measurements or (3) more than one tumor origin was known at time of MRI. Every patient was included only once, with the first cranial MRI containing at least one brain metastasis. Up to 10 metastases per patient were to be included. Brain metastases with large intra-tumoral hemorrhage (> 50% of the solid tumor part) were excluded.

### Test methods

A database search with the terms “cMRI” and (“NSCLC” or “SCLC” or “lung cancer”) was performed in our picture archiving and communication system (PACS) for patients scanned between 01/01/2009 and 01/01/2020. These patients were checked in our local tumor board for histological data and additional diseases.

### Image analysis

**Fig. 1:**
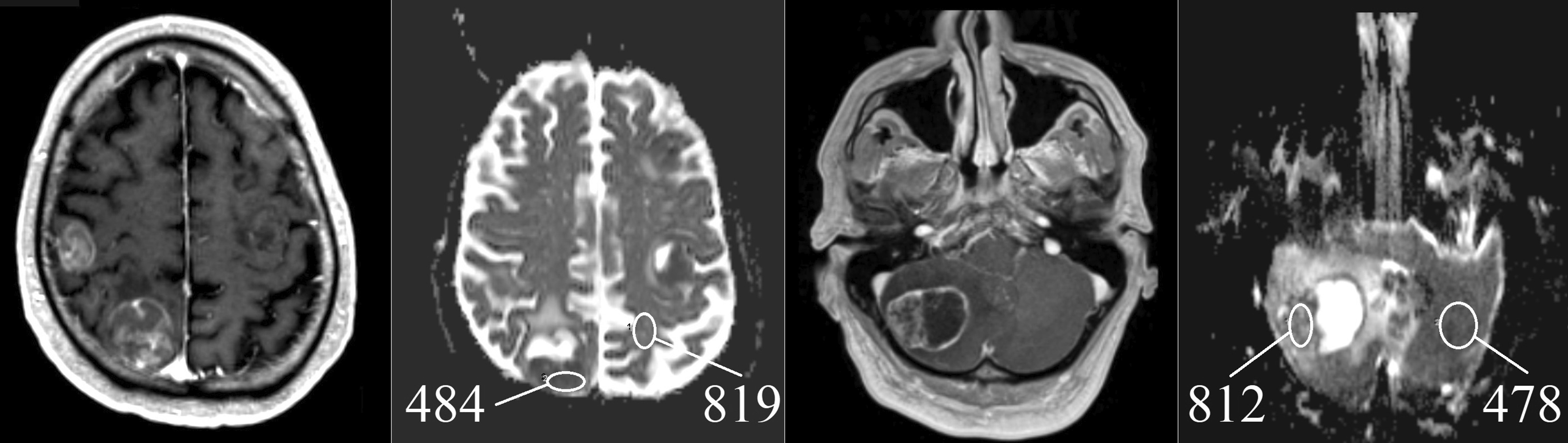
Two examples of ADC-ratios of brain metastasis in 3-T MRI (T1 with contrast agent and ADC map): left: typical SCLC with ADC ratio of 484/819 = 0.59, right: NSCLC with ADC ratio of 812/478 = 1.70. *SCLC* small cell lung cancer, *NSCLC* non-small cell lung cancer, *ADC* apparent diffusion coefficient

For comparing supra- and infra-tentorial metastases and different field strengths we developed a simple procedure. We measured the solid tumor part and the contralateral white matter and divided both values to calculate an ADC ratio, as shown in Fig. 1. A similar approach was used in a publication from Jung et al. [5] for comparing supra- and infratentorial brain metastases.

Two neuroradiology fellows (> 2 years’ experience in MRI diagnostics, blinded to clinical information) independently evaluated the cases and measured the ADC values. Another third (blinded) neuroradiologist evaluated 30 randomly chosen sample cases. The measuring method was explained with five sample cases. No further training was necessary.

Centricity™ PACS RA1000 Workstation (GE Healthcare, Chicago, ILLINOIS, USA) was used for measurements. The maximum diameter of the solid tumor area was determined on the enhanced T1 images combined with T2 and ADC maps. Finally, the corresponding area recorded in the ADC map was measured. The region of interest (ROI) was manually selected by each neuroradiologist separately. Adjacent large blood vessels and cystic necrosis should be avoided. The average ADC values was analyzed. In the event of a discrepancy (> 15% ADC ratio deviation), the case was discussed and examined for sources of error.

### Statistical analysis

The program Statistica, version 13 (TIBCO Software Inc., Palo Alto, CALIFORNIA, USA) was used. Significance level was set to *P* < 0.05. The comparison of ADC ratios of SCLC and NSCLC was performed using two sided Mann–Whitney U tests. Kruskal Wallis Test was used to compare ADC ratios of all different subtypes. Receiver Operating Curves (ROC) and Area Under Curve (AUC) values were used for calculating the ADC ratio with the highest diagnostic value to differentiate SCLC from NSCLC. The global optimum was determinated by maximizing Youden’s J. To test inter-rater reliability we calculated interclass correlation coefficient (ICC) estimates and their 95% confident intervals with R (packages irr, readxl, lpSolve and psych) based on mean-rating (k = 3), absolute-agreement, and a 2-way random-effects model.

## Results

### Participants

**Fig. 2:**
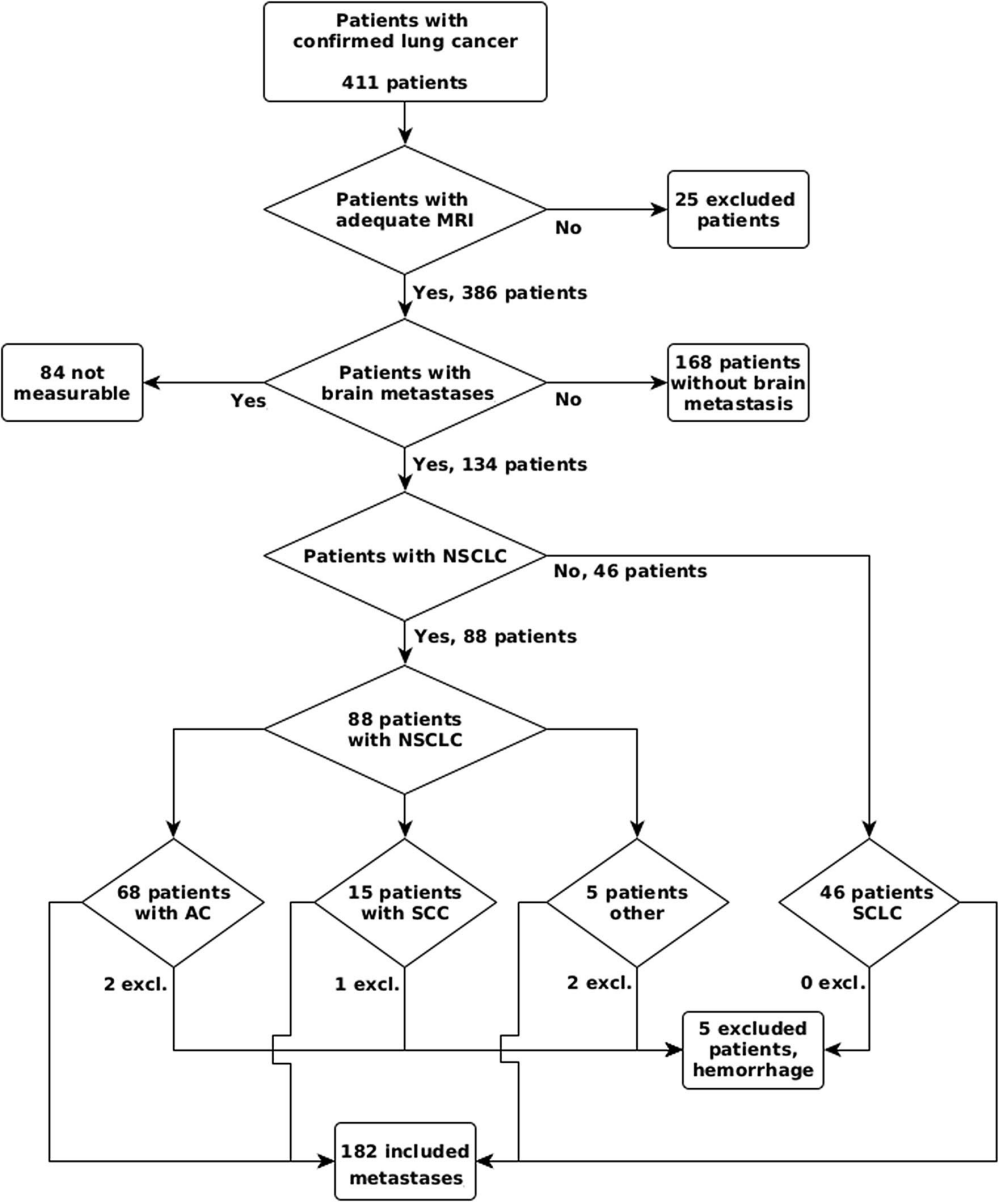
Participant flow chart. *AC* adenocarcinoma, *ADC* apparent diffusion coefficient, *CA* contrast agent, *NSCLC* non-small cell lung cancer, *SCC* squamous cell carcinoma, *SCLC* small cell lung cancer

**Fig. 3:**
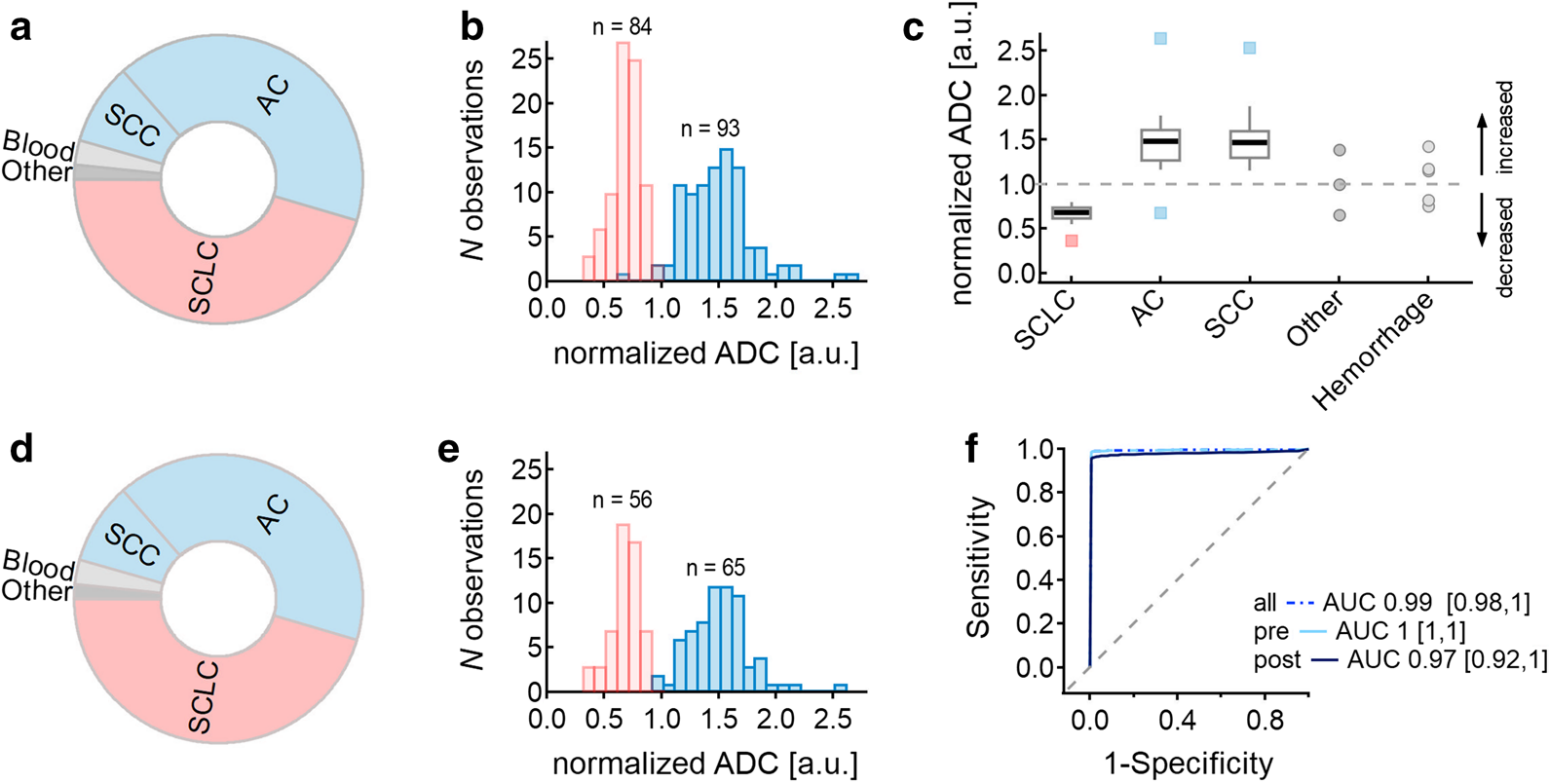
Overview results—**a** Pie charts of the patient cohort: distribution of patients with measurable brain metastases of lung cancer, and **d** distribution of only pre-therapeutic patients. **b** Histogram: distribution of ADC ratio of all measured metastases and, **e** only pre-therapeutic metastases of small cell lung cancer (left) and non-small cell lung cancer (right, contains only squamous cell carcinoma and adenocarcinoma). **c** Whisker-Box-Plot of ADC ratios grouped by histologies. **f** Receiver operating characteristics curve and area und curve of all, pre- and post-therapeutic patients with confidence interval. *AC* adenocarcinoma, *SCC* squamous cell carcinoma, *SCLC* small cell lung cancer

Our database contained 411 patients (162 females) with available imaging from 01.01.2009 to 01.01.2020. Mean age was 63.7 ± 9.5 years (mean ± standard deviation, range 31–89 years). Patients with confirmed SCLC were on average 62.9 ± 8.5 (mean ± standard deviation, range 40–83 years, n = 110, 40 females) years old. Figure 2 shows a flowchart of the patients with included brain metastases in this study. Finally, 129 patients were included in the analysis. Five patients with intra-tumoral hemorrhage were excluded from the main analysis but measured and analyzed for comparison purposes. The distribution of histologies did not differ significantly and is demonstrated in Fig. 3a, d.

### Interrater agreement

Inter rater reliability was good to excellent with an ICC estimate of 0.898 and a 95% confidence interval of 0.825 to 0.945 [9].

### MR Scanner and sequences

MRI data of six different MR scanners were included. ADC maps were based on echo-planar imaging DWI (EPI-DWI) in all cases, mostly with a slice thickness of 4 mm. Hence, ADC ratio is normalized and manufacturer and sequence independent, we abstain from a sequence/manufacturer specific evaluation, especially since our evaluated MRI were mainly performed on Siemens scanners, as shown in Table 1.

**Table 1:** MR scanners

Scanner	*N* (percent)	SCLC	AC	SCC	Other	Blood
3T-TIM Trio^1^	123 (66)	54	49	15	1	4
1.5T-AvantoFit^1^	41 (22)	20	18	1	1	1
3T-PrismaFit^1^	11 (6)	4	6	0	1	–
1.5T-Sonata^1^	6 (3)	3	2	1	–	–
3T-Discovery^2^	3 (2)	3	–	–	–	–
1.5T-Intera^3^	1 (1)	–	1	–	–	–

^1^Siemens

^2^GE Healthcare

^3^Philips

### Test results

#### SCLC

In 46 of 110 patients (32 of them without/before radiation or chemotherapy) with histologically confirmed small cell lung cancer 84 measurable brain metastases were detected. Initial diagnoses were made in 17 patients by brain biopsy, and in 29 patients via lung biopsy. 1.82 ± 1.16 metastases per patient (mean ± SD, range 1–6, median 1); in total 55 pre-therapeutic and 29 post-therapeutic brain metastases of patients with SCLC. Morphology of all SCLC metastases showed a distribution of 22% solid, 52% mixed cystic-solid and 26% cystic metastases.

Locations of metastases were in 11% of the patients only in the infratentorial and in 37% only in the supratentorial compartment. 52% of SCLC metastases were detected in both locations.

#### NSCLC

Sufficiently large brain metastases were found in 80 of 301 patients with confirmed NSCLC (73 of them without/before radiation or system therapy). First diagnoses were achieved in 21 patients by direct biopsy/resection of brain metastases, and in 59 cases by lung biopsy. Histology revealed in 66 cases adenocarcinomas (AC), and in 14 squamous cell carcinomas (SCC). Overall 93 NSCLC metastases (1.19 ± 0.44 metastases per patient, range 1–3, median 1), a total of 71 pre-therapeutic and 22 post-therapeutic values, were analyzed. Morphology contained 37% solid, 40% mixed cystic-solid and 23% cystic metastases.

In 12% (AC 12%, SCC 14%) of the patients only the infratentorial and in 49% (AC 45%, SCC 64%) only the supratentorial compartment was affected. In 39% (AC 43%, SCC 22%) NSCLC metastases were detected in both locations.

#### Other

Three patients had neither AC nor SCC, i.e. two had large cell neuroendocrine tumor, and one had signet ring cell carcinoma. Five patients with hemorrhage of brain metastases were separately analyzed.

#### Differentiation of SCLC and NSCLC

The mean ADC ratios of brain metastasis were 0.68 ± 0.12 for SCLC (n = 84), and 1.47 ± 0.31 for NSCLC. A histogram of the distribution of ADC ratios of all measured metastases is shown in Fig. 3b.

The two sided Mann–Whitney U tests showed a significant differentiation between SCLC and NSCLC (*p* < 0.001). Youden’s J analyses of AUC estimated ADC ratio < 0.97 being the optimal cut-off value with a sensitivity of 99% (83/84) and specificity of 99% (92/93) for detecting SCLC, as shown in Fig. 3f. An AD ratio of 0.98 shows a sensitivity of 100% and specificity of 97% (90/93). Figure 3c demonstrates a box-plot of the four subgroups and the excluded intratumoral hemorrhage.

#### Pre-therapeutic differentiation of SCLC and NSCL

In 100% of the pre-therapeutic SCLC cases, the ADC ratio was 0.9 or below (0.66 ± 0.12 for the solid part of the metastasis). ADC ratio (NSCLC) was 1.50 ± 0.30. ADC ratios between 0.89 and 0.96 showed a sensitivity and specificity of 100% in detecting SCLC (n = 55) versus NSCLC (n = 71, 58 AC and 13 SCC); pre-therapeutic, without hemorrhage and with either AC or SCC histology. Only one (in this analysis excluded) pre-therapeutic not-SCLC metastasis (of a large cell neuroendocrine tumor) showed an ADC ratio < 0.9. Figure 3e demonstrates the distribution of measured ADC ratios in a histogram. The two sided Mann–Whitney U tests stated a significant differentiation between SCLC and NSCLC (*p* < 0.001).

#### Post-therapeutic ADC ratio

Post-therapeutic brain metastases (29 SCLC, 18 AC, 4 SCC) showed an average ADC ratio of 0.70 ± 0.13 for SCLC, and 1.37 ± 0.34 for NSCLC. The two sided Mann–Whitney U tests still showed a significant differentiation between SCLC and NSCLC (*p* < 0.001). RoC and AuC analyses estimated ADC ratio < 0.97 being the optimal cut-off value with a sensitivity of 100% and specificity of 96% (23/24) for detecting SCLC.

#### Subgroup analysis

Separate analysis for AC and SCC demonstrated slightly smaller ADC ratios for AC but no significant differences, as demonstrated in Table 2. Two-tailed Kruskal Wallis Test of the four different subtypes and intratumoral hemorrhage showed significant differences between (1) SCLC and AC and (2) SCLC and SCC, but no significant differences between the other groups, as shown in Additional file 2: Table S1.

**Table 2:** ADC ratio subtype analyses of brain metastases

Subtype	*N*	Mean	Min	Max	*SD*
SCLC	84	0.68	0.36	0.97	0.12
Pre-therapeutic	55	0.66	0.36	0.88	0.12
Post-therapeutic	29	0.70	0.47	0.97	0.13
AC	76	1.47	0.68	2.64	0.30
Pre-therapeutic	58	1.50	1.00	2.64	0.28
Post-therapeutic	18	1.37	0.68	2.15	0.36
SCC	17	1.52	0.97	2.53	0.36
Pre-therapeutic	13	1.56	0.97	2.53	0.39
Post-therapeutic	4	1.38	1.10	1.66	0.25
Other	3	1.01	0.65	1.39	0.37
Hemorrhage	5	1.06	0.75	1.42	0.28

*SCLC* small cell lung cancer, *AC* adenocarcinoma, *SCC* squamous cell carcinoma

## Discussion

Our study in 411 patients with lung cancer demonstrates the importance of pre-therapeutic cranial MRI, which is still a matter of debate [10, 11], even if neurologic metastases [12] are common in lung cancer. Following the results of our study, we can distinguish SCLC from NSCLC on initial diagnostic work-up.

Although brain metastases are improbable in early stage NSCLC without central neurologic symptoms [13], the risk of brain metastasis, especially in SCLC is omnipresent [14]. Large studies show bad survival rates for patients with limited [15] and extended disease [16–18]. Even if new system therapies have increased to mean survival time compared to old studies [19], an early and effective therapy seems to be an important factor in limited disease [20, 21]. Hence, even in extended diseases, fast diagnosis and therapy is important [22–24]. The prognosis remains poor, although the combination of chemotherapy with new immune therapies seems to improve the survival [25, 26]. In contrast to SCLC the more common AC [27] and SCC [28] show a slightly bettering of the long time survival rate.

Thus an additional surgery can influence the course of the disease [29] as well as a complicated wound healing [3]. Radiosurgery and whole brain therapy are possible alternatives to surgery [30, 31].

“Nomen est omen”, but it has long been known that the average cytoplasmic diameter of SCLC is significantly lower than NSCLC (*p* < 0.0001), with approx. 8.4 microns versus 15 microns, as shown by Vollmer [6]. A small study from Korea could not show a significant lower diffusion restriction using an ADC-ratio-like approach, but a correlation with epidermal growth factor receptor (EGFR) expression [5]. A recent study from Turkey showed a possible differentiation of SCLC and AC using ADC-Histograms [7]. Compared to other studies more patients with SCLC and less with SCC were included in our study. The mean ADC ratio of SCC and AC was higher than in other studies and the differentiation between SCLC and NSCLC was clearer. ADC ratios of tumor cells are multifactorial influenced, e.g. by irregular angiogenesis or abnormal extracellular matrix [32, 33], and did not only depend on cell diameters. Although the sensitivity and specificity in our study seems to be astonishing high. Mainly, because the low cell diameter seems to be the dominating factor in pre-therapeutic SCLC, which leads to a lowering of the ADC.

Localization and morphology of brain metastases were similar to other studies [34, 35].

The occurrence of brain metastases in our cohort was significantly higher (56% at all, for pre-therapeutic patients with (measureable) brain metastases: 31% SCLC, 25% NSCLC, respectively) than in other studies [1, 36]. We only included patients with neuroradiologic imaging, which leads bias towards potentially neurologic strongly affected patients with a higher probability of brain metastases.

### Limitations

Measurement of solid tumor parts of metastases keeps a challenging task. The wrong selection of tumor edema, cystic parts or blood can strongly influence the ADC ratio. For this reason, we separately controlled the measurements by two neuroradiology fellows. A third (blinded) neuroradiologist repeated the measurement for 30 randomly chosen cases, and again a good to excellent interrater reliability was demonstrated.

Visual features of metastasis allow a clear differentiation of the solid tumor portion, if enhanced T1-, T2- and DWI sequences are viewed in parallel, each with a sufficient layer thickness (≤ 4 mm) and with the same orientation (e.g. transversal).

Even if in cases with heterogeneous solid tumor parts small differences in the measurements were noticed, the inter-rater-reliability was sufficient. Nevertheless, an objective procedure for selecting the region of interest should be evaluated, which is needed to establish this method into clinical routine.

The count of post-therapeutic measurable metastases was low, wherefore we did not perform a subgroup analysis for chemotherapy and radiotherapy. Although, we noticed a greater variance in the measured values.

### Outlook

Prospective data should be collected and analyzed to confirm our findings. The extent of resection of a metastasis beyond the incision margins is still unclear, here DWI sequences can reveal additional information [37]. Metastases after radiation and/or chemotherapy showed an different ADC ratio, which may be used for therapy monitoring [38–40].

## Conclusion

In pre-therapeutic patients with lung cancer and brain metastases with solid tumor parts, ADC ratio enables an excellent differentiation of SCLC and NSCLC.

## Supplementary Information

**Additional file 1.** STARD 2015 protocol.

**Additional file 2.** Kruskal Wallis Test results for the differentiation of lung cancer subtypes using ADC-ratio.

## Data Availability

The datasets used and analyzed during the current study are available from the corresponding author on reasonable request.

## Abbreviations

AC: Adeno carcinoma
ADC: Apparent diffusion coefficient
AUC: Area under curve
cMRI: Cranial magnetic resonance imaging
DWI: Diffusion weighted imaging
ICC: Interclass correlation coefficient
MR: Magnetic resonance
NSCLC: Non-small cell lung cancer
ROC: Receiver operating characteristics
SCC: Squamous cell carcinoma
SCLC: Small cell lung cancer
SD: Standard deviation
