# Supplementary material for: Differentiation of brain metastases from small and non-small lung cancers using apparent diffusion coefficient (ADC) maps
Source: BMC Med Imaging. 2021 Apr 15;21:70. doi: 10.1186/s12880-021-00602-7 (PMC8048287; doi:10.1186/s12880-021-00602-7)
Supplement: Supplementary file 2 — Additional file 2. Kruskal Wallis Test results for the differentiation of lung cancer subtypes using ADC-ratio. [file 12880_2021_602_MOESM2_ESM.pdf]

Diagnostic value of apparent diffusion coefficient maps for the differentiation of brain metastases from lung cancer

Sebastian Müller; Eya Khadhraoui; Nicole Neef; Marielle Ernst; Christian Riedel

Supplementary material

Supplementary Table 1 – Kruskal Wallis Test results of brain metastases. Multiple comparisons p values (2-tailed).

	**SCLC**<br>rank sum 44<br>*p*-value	**AC**<br>rank sum 137<br>*p*-value	**SCC**<br>rank sum 140<br>*p*-value	**Other**<br>rank sum 85<br>*p*-value	**Hemorrhage**<br>rank sum 95<br>*p*-value
**SCLC**		**0.0001**	**0.0001**	1.00	0.39
**AC**	**<0.0001**		p=1.00	0.96	0.89
**SCC**	**<0.0001**	1.00		1.00	1.00
**Other**	1.00	0.96	1.00		1.00
**Hemorrhage**	0.39	0.89	1.00	1.00	

Table legend: SCLC small cell lung cancer, AC adenocarcinoma, SCC squamous cell carcinoma.
